# 5-Bromo-1*H*-pyrrolo­[2,3-*b*]pyridine

**DOI:** 10.1107/S1600536813004157

**Published:** 2013-02-16

**Authors:** Pavel Štarha, Zdeněk Trávníček

**Affiliations:** a Department of Inorganic Chemistry, Faculty of Science, Palacký University, 17. listopadu 12, CZ-771 46 Olomouc, Czech Republic

## Abstract

In the title compound, C_7_H_5_BrN_2_, fused six-membered pyridine and five-membered pyrrole rings form the essentially planar aza­indole skeleton (r.m.s. deviation = 0.017 Å). In the crystal, pairs of N—H⋯N hydrogen bonds connect the mol­ecules into inversion dimers.

## Related literature

For the structure of 7-aza­indole (C_7_H_6_N_2_), see: Dufour *et al.* (1990 ▶) and for the structure of 3-iodo-7-aza­indole (C_7_H_5_IN_2_), see: Chou *et al.* (2000 ▶). For the utilization of the title compound as the N-donor carrier ligand of highly cytotoxic platinum(II) dichlorido complexes, see: Štarha *et al.* (2012 ▶).
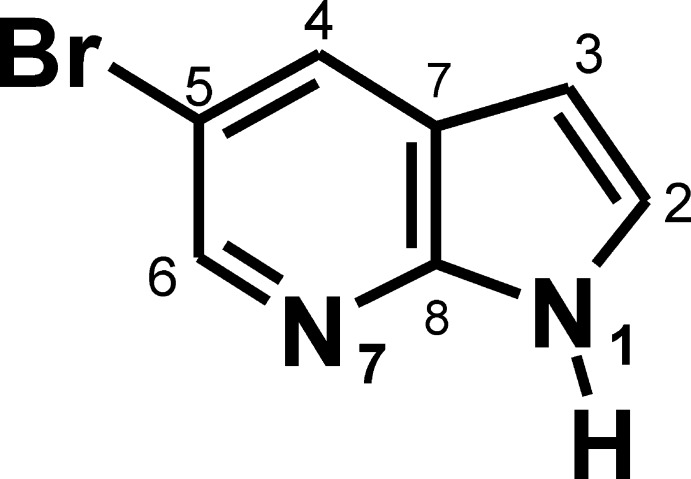


## Experimental

### 

#### Crystal data

C_7_H_5_BrN_2_
*M* *_r_* = 197.04
Monoclinic, 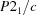

*a* = 8.9082 (4) Å
*b* = 13.3632 (6) Å
*c* = 5.8330 (3) Å
β = 103.403 (5)°
*V* = 675.47 (6) Å^3^
*Z* = 4
Mo *K*α radiation
μ = 6.00 mm^−1^
*T* = 100 K
0.24 × 0.24 × 0.12 mm

#### Data collection

Oxford Diffraction Xcalibur Sapphire2 CCD diffractometer
Absorption correction: multi-scan (*CrysAlis RED*; Oxford Diffraction, 2009 ▶) *T*_min_ = 0.327, *T*_max_ = 0.533
3977 measured reflections
1185 independent reflections
1047 reflections with *I* > 2σ(*I*)
*R*_int_ = 0.022

#### Refinement

*R*[*F* ^2^ > 2σ(*F* ^2^)] = 0.035
*wR*(*F* ^2^) = 0.092
*S* = 1.13
1185 reflections
91 parameters
H-atom parameters constrained
Δρ_max_ = 1.69 e Å^−3^
Δρ_min_ = −0.33 e Å^−3^

### 

Data collection: *CrysAlis CCD* (Oxford Diffraction, 2009 ▶); cell refinement: *CrysAlis RED* (Oxford Diffraction, 2009 ▶); data reduction: *CrysAlis RED*; program(s) used to solve structure: *SHELXS97* (Sheldrick, 2008 ▶); program(s) used to refine structure: *SHELXL97* (Sheldrick, 2008 ▶); molecular graphics: *DIAMOND* (Brandenburg *et al.*, 2011 ▶); software used to prepare material for publication: *publCIF* (Westrip, 2010 ▶).

## Supplementary Material

Crystal structure: contains datablock(s) I, global. DOI: 10.1107/S1600536813004157/tk5196sup1.cif

Structure factors: contains datablock(s) I. DOI: 10.1107/S1600536813004157/tk5196Isup2.hkl

Supplementary material file. DOI: 10.1107/S1600536813004157/tk5196Isup3.cml

Additional supplementary materials:  crystallographic information; 3D view; checkCIF report

## Figures and Tables

**Table 1:** Hydrogen-bond geometry (Å, °)

*D*—H⋯*A*	*D*—H	H⋯*A*	*D*⋯*A*	*D*—H⋯*A*
N1—H1⋯N7^i^	0.88	2.12	2.960 (5)	159

Symmetry code: (i) 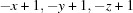.

**Fig. 1.**
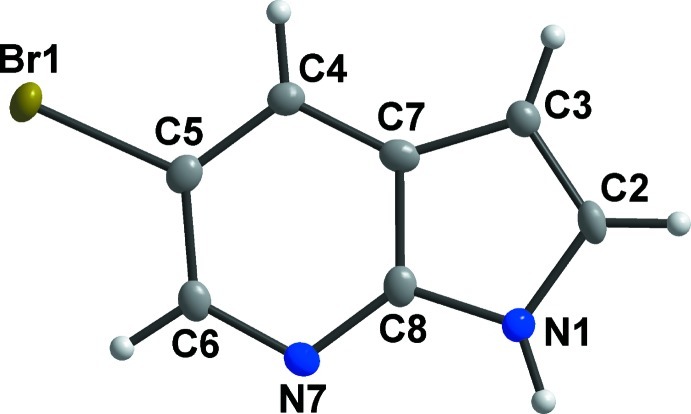
The molecular structure of the title compound with the non-hydrogen atoms depicted as displacement ellipsoids at the 50% probability level and given with the atom numbering scheme.

**Fig. 2.**
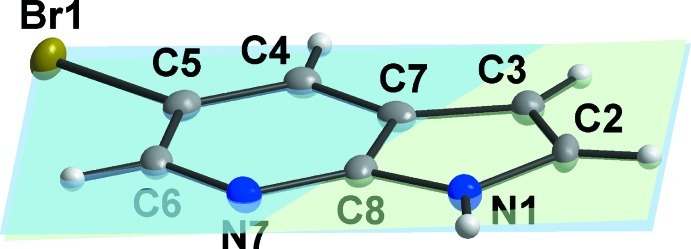
A view of the title compound showing the mutual orientation of the six-membered pyridine (least-squares plane created through the C4, C5, C6, N7, C8 and C7 atoms; in blue) and five-membered pyrrole rings (least-squares plane created through the N1, C2, C3, C7 and C8 atoms; in green). The planes are nearly coplanar forming the dihedral angle of 2.09 (14)°.

**Fig. 3.**
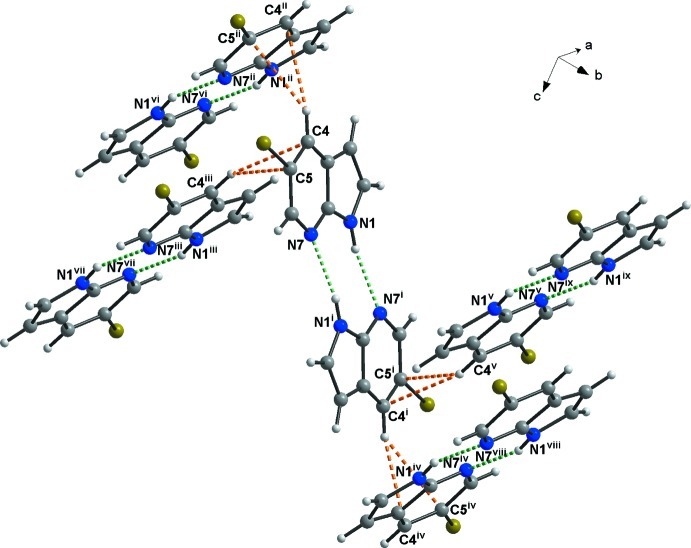
Part of the crystal structure of the title compound (ball-and-stick model) showing the N—H···N hydrogen bonds (dashed green lines) and C—H···C non-covalent contacts (dashed orange lines). [Symmetry codes: (i) 1 - x, 1 - y, 1 - z; (ii) x, 0.5 - y, z - 0.5; (iii) x, 0.5 - y, z + 0.5; (iv) 1 - x, y + 0.5, 1.5 - z; (v) 1 - x, y + 0.5, 0.5 - z; (vi) 1 - x, y - 0.5, 0.5 - z; (vii) 1 - x, y - 0.5, 1.5 - z; (viii) x, 1.5 - y, z + 0.5; (ix) x, 1.5 - y, z - 0.5].

**Fig. 4.**
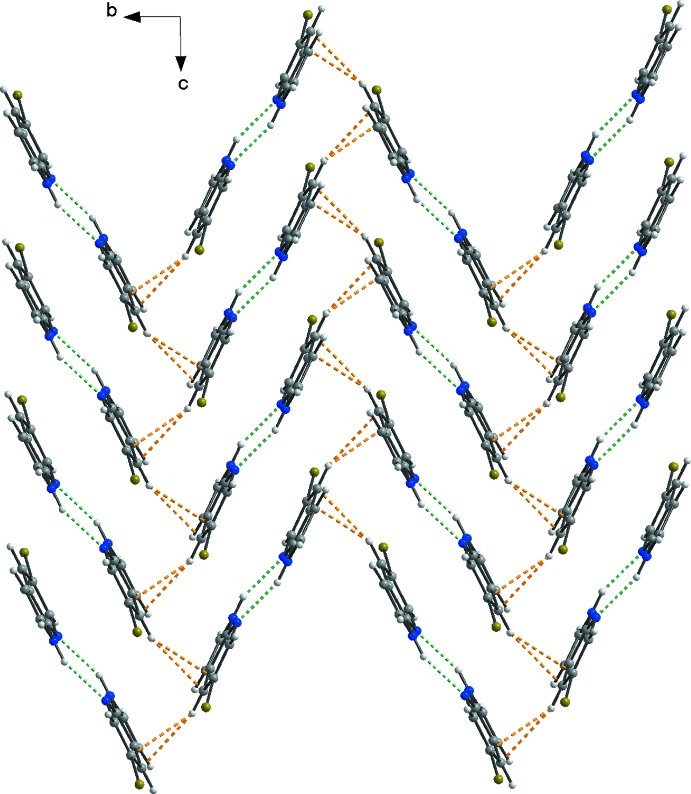
Part of the crystal structure of the title compound (ball-and-stick model) showing the formation of zigzag chains; dashed green lines indicate the N—H···N hydrogen bonds and dashed orange lines indicate C—H···C non-covalent contacts.

## supplementary crystallographic information

### Comment

The title compound 5-bromo-7-azaindole (5BrHaza), which is commercially available, was recently used, together with 3-chloro-7-azaindole and 3-iodo-7-azaindole, for the preparation of the platinum(II) dichlorido and oxalato (ox) complexes of the general formula *cis*-[PtCl_2_(nHaza)_2_], and [Pt(ox)(nHaza)_2_], respectively (Štarha *et al.*, 2012); nHaza stands for the above-mentioned 7-azaindole halogeno-derivatives. The prepared Pt(II)-dichlorido complexes were found to be highly cytotoxic against the osteosarcoma (HOS), breast carcinoma (MCF7) and prostate carcinoma (LNCaP) human cancer cell lines. Particularly *cis*-[PtCl_2_(5BrHaza)_2_] exceeded the clinically applied platinum-based anticancer drug cisplatin, (*cis*-[PtCl_2_(NH_3_)_2_]), since its IC_50_ values (the concentration lethal for 50% of the tested cancer cells) equaled 2.5 µM (HOS; 34.2 µM for cisplatin), 2.0 µM (MCF7; 19.6 µM for cisplatin) and 1.5 µM (LNCaP; 3.8 µM for cisplatin).

The discrete molecules (Fig. 1) of the title compound contain fused six-membered pyridine and five-membered pyrrole rings forming the 7-azaindole skeleton. The planes fitted through the atoms of both rings form a dihedral angle of 2.09 (14)° (Fig. 2). The most deviated atoms from the mentioned planes are C6 (-0.012 (4) Å) and C3 (-0.006 (4) Å), respectively, while the most deviated atom from the plane fitted through nonhydrogen atoms of the 7-azaindole moiety is C5 (0.025 (4) Å).

The crystal structure contains the N—H···N hydrogen bonds and C—H···C non-covalent contacts (Fig. 3). Two N1—H1···N7 hydrogen bonds (Table 1) bind together two 5BrHaza molecules into a centrosymmetric dimer. The dimers are connected by C4—H4···C4 and C4—H···C5 non-covalent contacts (see Hydrogen-bond geometry) with four other dimers, which results in the formation of a 2D supramolecular array (Fig. 4).

The molecular structure of the title compound resembles literature precedents: 7-azaindole (Dufour *et al.*, 1990) and 3-iodo-7-azaindole (Chou *et al.*, 2000).

### Experimental

The title compound was employed as a starting compound of the syntheses of *cis*-[PtCl_2_(5BrHaza)_2_] and [Pt(ox)(5BrHaza)_2_].0.75H_2_O (Štarha *et al.*, 2012). These complexes were prepared by the reactions of the appropriate platinum(II) salt (K_2_[PtCl_4_] or K_2_[Pt(ox)_2_].2H_2_O; water solution, 0.5 mmol) with 1.0 mmol of 5BrHaza dissolved in ethanol at 50 °C. Microcrystals obtained as a main product during 2 days of stirring were filtered off and the filtrate was left to crystalize at laboratory temperature in the case of the oxalato-5BrHaza complex. The colourless crystals, which formed as a by-product during a slow evaporation in next 2 weeks, were collected and characterized by elemental analysis, NMR spectroscopy and single-crystal X-ray analysis. ^1^H NMR (DMF-*d_7_*, TMS, 298 K, p.p.m.): *δ* 11.91 (bs, 1H, HN^1^), 8.30 (d, *J* = 2.2 Hz, 1H, HC^6^), 8.20 (d, *J* = 2.0 Hz, 1H, HC^4^), 7.63 (t, *J* = 2.8 Hz, 1H, HC^2^), 6.50 (m, 1H, HC^3^). ^13^C NMR (DMF-*d_7_*, TMS, 298 K, p.p.m.): *δ* 147.5 (C^8^), 142.9 (C^6^), 130.3 (C^4^), 128.2 (C^2^), 122.1 (C^7^), 111.1 (C^5^), 100.0 (C^3^). ^15^N NMR (DMF-*d_7_*, relative to DMF, 298 K, p.p.m.): *δ* 140.9 [s, 11.93, HN^1^; s, 7.63, HC^2^; s, 6.50, HC^3^ (N^1^)], 277.5 [s, 8.30, HC^6^ (N^7^)]. Analysis calculated for C_7_H_6_BrN_2_: C 42.67, H 2.56, N 14.22%; found: C 42.58, H 2.62, N 14.09%. Elemental analysis (C, H, N) was performed on a Thermo Scientific Flash 2000 CHNO-S Analyzer. The ^1^H, ^13^C and ^15^N NMR spectra of the DMF-*d_7_* solutions were collected at 300 K on a Varian 400 spectrometer at 400.00 MHz, 100.58 MHz and 40.53 MHz, respectively. ^1^H and ^13^C spectra were calibrated using tetramethylsilane (TMS) as a reference. The ^15^N NMR spectrum was measured relative to the DMF signals.

### Refinement

Hydrogen atoms were located in difference maps and refined using the riding model with C—H = 0.95 Å and N—H = 0.88 Å, and with *U*_iso_(H) = 1.2*U*_eq_(CH, NH). The maximum and minimum residual electron density peaks of 1.69 and -0.33 eÅ^-3^, respectively, were located 1.72 Å and 1.25 Å from the H6 and C6 atoms, respectively.

### Crystal data

**Table:**

C_7_H_5_BrN_2_	*F*(000) = 384
*M**_r_* = 197.04	*D*_x_ = 1.938 Mg m^−^^3^
Monoclinic, *P*2_1_/*c*	Mo *K*α radiation, λ = 0.71073 Å
Hall symbol: -P 2ybc	Cell parameters from 4062 reflections
*a* = 8.9082 (4) Å	θ = 3.0–33.1°
*b* = 13.3632 (6) Å	µ = 6.00 mm^−^^1^
*c* = 5.8330 (3) Å	*T* = 100 K
β = 103.403 (5)°	Prim, colourless
*V* = 675.47 (6) Å^3^	0.24 × 0.24 × 0.12 mm
*Z* = 4	

### Data collection

**Table:**

Oxford Diffraction Xcalibur Sapphire2 CCD diffractometer	1185 independent reflections
Radiation source: Enhance (Mo) X-ray Source	1047 reflections with *I* > 2σ(*I*)
Graphite monochromator	*R*_int_ = 0.022
Detector resolution: 8.3611 pixels mm^-1^	θ_max_ = 25.0°, θ_min_ = 3.1°
ω scans	*h* = −10→9
Absorption correction: multi-scan (*CrysAlis RED*; Oxford Diffraction, 2009)	*k* = −15→15
*T*_min_ = 0.327, *T*_max_ = 0.533	*l* = −6→6
3977 measured reflections	

### Refinement

**Table:**

Refinement on *F*^2^	Primary atom site location: structure-invariant direct methods
Least-squares matrix: full	Secondary atom site location: difference Fourier map
*R*[*F*^2^ > 2σ(*F*^2^)] = 0.035	Hydrogen site location: inferred from neighbouring sites
*wR*(*F*^2^) = 0.092	H-atom parameters constrained
*S* = 1.13	*w* = 1/[σ^2^(*F*_o_^2^) + (0.0578*P*)^2^ + 1.0071*P*] where *P* = (*F*_o_^2^ + 2*F*_c_^2^)/3
1185 reflections	(Δ/σ)_max_ < 0.001
91 parameters	Δρ_max_ = 1.69 e Å^−^^3^
0 restraints	Δρ_min_ = −0.33 e Å^−^^3^

### Special details

**Table:**

Geometry. All e.s.d.'s (except the e.s.d. in the dihedral angle between two l.s. planes) are estimated using the full covariance matrix. The cell e.s.d.'s are taken into account individually in the estimation of e.s.d.'s in distances, angles and torsion angles; correlations between e.s.d.'s in cell parameters are only used when they are defined by crystal symmetry. An approximate (isotropic) treatment of cell e.s.d.'s is used for estimating e.s.d.'s involving l.s. planes.
Refinement. Refinement of *F*^2^ against ALL reflections. The weighted *R*-factor *wR* and goodness of fit *S* are based on *F*^2^, conventional *R*-factors *R* are based on *F*, with *F* set to zero for negative *F*^2^. The threshold expression of *F*^2^ > σ(*F*^2^) is used only for calculating *R*-factors(gt) *etc*. and is not relevant to the choice of reflections for refinement. *R*-factors based on *F*^2^ are statistically about twice as large as those based on *F*, and *R*- factors based on ALL data will be even larger.

### Fractional atomic coordinates and isotropic or equivalent isotropic displacement parameters (Å^2^)

**Table:**

	*x*	*y*	*z*	*U*_iso_*/*U*_eq_	
Br1	−0.02317 (5)	0.34425 (3)	−0.26611 (7)	0.0188 (2)	
N1	0.6033 (4)	0.4228 (3)	0.3167 (6)	0.0151 (7)	
H1	0.6364	0.4519	0.4545	0.018*	
C2	0.6944 (5)	0.3858 (3)	0.1738 (8)	0.0168 (9)	
H2	0.8039	0.3886	0.2096	0.020*	
C3	0.6054 (5)	0.3447 (3)	−0.0253 (8)	0.0151 (9)	
H3	0.6411	0.3135	−0.1491	0.018*	
C4	0.3052 (5)	0.3375 (3)	−0.1574 (7)	0.0143 (9)	
H4	0.2942	0.3057	−0.3060	0.017*	
C5	0.1780 (5)	0.3665 (3)	−0.0748 (7)	0.0153 (9)	
C6	0.1933 (5)	0.4118 (3)	0.1439 (7)	0.0150 (9)	
H6	0.1023	0.4281	0.1944	0.018*	
N7	0.3299 (4)	0.4335 (2)	0.2865 (6)	0.0153 (7)	
C7	0.4494 (5)	0.3572 (3)	−0.0129 (7)	0.0148 (9)	
C8	0.4527 (5)	0.4061 (3)	0.2052 (7)	0.0150 (9)	

### Atomic displacement parameters (Å^2^)

**Table:**

	*U*^11^	*U*^22^	*U*^33^	*U*^12^	*U*^13^	*U*^23^
Br1	0.0135 (3)	0.0203 (3)	0.0197 (3)	0.00003 (15)	−0.00191 (18)	−0.00174 (16)
N1	0.0141 (19)	0.0143 (16)	0.0167 (18)	−0.0019 (13)	0.0034 (15)	−0.0021 (14)
C2	0.011 (2)	0.015 (2)	0.025 (2)	0.0024 (16)	0.0054 (18)	0.0023 (18)
C3	0.015 (2)	0.013 (2)	0.018 (2)	0.0006 (15)	0.0045 (17)	0.0006 (16)
C4	0.018 (2)	0.010 (2)	0.015 (2)	−0.0014 (15)	0.0040 (18)	0.0003 (15)
C5	0.017 (2)	0.010 (2)	0.018 (2)	−0.0010 (15)	0.0015 (18)	0.0021 (15)
C6	0.014 (2)	0.010 (2)	0.022 (2)	0.0001 (15)	0.0050 (17)	0.0028 (16)
N7	0.0166 (18)	0.0121 (16)	0.0179 (18)	−0.0007 (13)	0.0057 (15)	−0.0016 (14)
C7	0.021 (2)	0.0087 (19)	0.016 (2)	0.0007 (15)	0.0063 (18)	0.0006 (15)
C8	0.014 (2)	0.010 (2)	0.021 (2)	−0.0002 (15)	0.0032 (17)	0.0033 (16)

### Geometric parameters (Å, º)

**Table:**

Br1—C5	1.902 (4)	C4—C5	1.385 (6)
N1—C8	1.367 (6)	C4—C7	1.389 (6)
N1—C2	1.383 (5)	C4—H4	0.9500
N1—H1	0.8800	C5—C6	1.390 (6)
C2—C3	1.361 (6)	C6—N7	1.337 (5)
C2—H2	0.9500	C6—H6	0.9500
C3—C7	1.418 (6)	N7—C8	1.339 (5)
C3—H3	0.9500	C7—C8	1.425 (6)
			
C8—N1—C2	107.6 (4)	C4—C5—Br1	119.2 (3)
C8—N1—H1	126.2	C6—C5—Br1	119.0 (3)
C2—N1—H1	126.2	N7—C6—C5	123.1 (4)
C3—C2—N1	110.6 (4)	N7—C6—H6	118.4
C3—C2—H2	124.7	C5—C6—H6	118.4
N1—C2—H2	124.7	C6—N7—C8	114.9 (3)
C2—C3—C7	107.0 (4)	C4—C7—C3	136.6 (4)
C2—C3—H3	126.5	C4—C7—C8	116.9 (4)
C7—C3—H3	126.5	C3—C7—C8	106.4 (4)
C5—C4—C7	116.9 (4)	N7—C8—N1	125.4 (4)
C5—C4—H4	121.5	N7—C8—C7	126.3 (4)
C7—C4—H4	121.5	N1—C8—C7	108.3 (4)
C4—C5—C6	121.8 (4)		

### Hydrogen-bond geometry (Å, º)

**Table:**

*D*—H···*A*	*D*—H	H···*A*	*D*···*A*	*D*—H···*A*
N1—H1···N7^i^	0.88	2.12	2.960 (5)	159
C4—H4···C4^ii^	0.95	2.82	3.738 (6)	162
C4—H4···C5^ii^	0.95	2.84	3.656 (6)	144

Symmetry codes: (i) −*x*+1, −*y*+1, −*z*+1; (ii) *x*, −*y*+1/2, *z*−1/2.

## References

[bb1] Brandenburg, K. (2011). *DIAMOND* Crystal Impact GbR, Bonn, Germany.

[bb2] Chou, P. T., Liao, J. H., Wei, C. Y., Yang, C. Y., Yu, W. S. & Chou, Y. H. (2000). *J. Am. Chem. Soc.* **122**, 986–987.

[bb3] Dufour, P., Dartiguenave, Y., Dartiguenave, M., Dufour, N., Lebuis, A. M., Belanger-Gariepy, F. & Beauchamp, A. L. (1990). *Can. J. Chem.* **68**, 193–202.

[bb4] Oxford Diffraction (2009). *CrysAlis CCD* and *CrysAlis RED* Oxford Diffraction Ltd, Yarnton, England.

[bb5] Sheldrick, G. M. (2008). *Acta Cryst.* A**64**, 112–122. 10.1107/S0108767307043930 18156677

[bb6] Štarha, P., Trávníček, Z., Popa, A., Popa, I., Muchová, T. & Brabec, V. (2012). *J. Inorg. Biochem.* **115**, 57–63. 10.1016/j.jinorgbio.2012.05.006 22922312

[bb7] Westrip, S. P. (2010). *J. Appl. Cryst.* **43**, 920–925.

